# A geographical cluster randomised stepped wedge study of continuing medical education and cancer diagnosis in general practice

**DOI:** 10.1186/s13012-014-0159-z

**Published:** 2014-11-07

**Authors:** Berit Skjødeberg Toftegaard, Flemming Bro, Peter Vedsted

**Affiliations:** Research Unit for General Practice, Aarhus University, Bartholins Allé 2, DK-8000 Aarhus, Denmark; Research Centre for Cancer Diagnosis in Primary Care (CaP), Aarhus University, Bartholins Allé 2, DK-8000 Aarhus, Denmark; Department of Public Health, Section for General Medical Practice, Bartholins Allé 2, DK-8000 Aarhus, Denmark

**Keywords:** Denmark, General Practice, Continuing Medical Education, Diagnosis, Early Detection of Cancer, Barriers

## Abstract

**Background:**

Denmark has inferior cancer survival rates compared with many European countries. The main reason for this is suggested to be late diagnosis at advanced cancer stages. Cancer diagnostic work-up begins in general practice in 85% of all cancer cases. Thus, general practitioners (GPs) play a key role in the diagnostic process. The latest Danish Cancer Plan included continuing medical education (CME) on early cancer diagnosis in general practice to improve early diagnosis. This dual aims of this protocol are, first, to describe the conceptualisation, operationalisation and implementation of the CME and, second, to describe the study design and outcomes chosen to evaluate the effects of the CME.

**Methods/Design:**

The intervention is a CME in early cancer diagnosis targeting individual GPs. It was developed by a step-wise approach. Barriers for early cancer diagnosis at GP level were identified systematically and analysed using the behaviour system involving capability, opportunity and motivation described by Michie et al. The study will be designed as a geographical cluster randomised stepped wedge study. The study population counts 836 GPs from 417 general practices in the Central Denmark Region, geographically divided into eight clusters. GPs from each cluster will be invited to a CME meeting at a certain date three weeks apart. The primary outcomes will be primary care interval and GP referral rate on cancer suspicion. Data will be obtained from national registries, GP-completed forms on patients referred to cancer fast-track pathways and GP-completed online questionnaires before and after the intervention.

**Discussion:**

To our knowledge, this will be the first study to measure the effect of a theory-based CME in early cancer diagnosis at three levels: GP knowledge and attitude, GP activity and patient outcomes. The achieved knowledge will contribute to the understanding of whether and how general practice’s ability to perform cancer diagnosis may be improved.

**Trial registration:**

Registered as NCT02069470 on ClinicalTrials.gov.

**Electronic supplementary material:**

The online version of this article (doi:10.1186/s13012-014-0159-z) contains supplementary material, which is available to authorized users.

## Background

Denmark lags behind many European countries in cancer survival. It has been suggested that late diagnosis and more advanced cancer stage at treatment lie at the root of this problem [1-9]. In order to minimise the time from referral to diagnosis, some countries have implemented fast-track pathways for cancer, e.g. the 2-week wait in the United Kingdom and the cancer pathways in Denmark [10,11]. Such organisational initiatives are largely dependent on the prevailing referral patterns in general practice. Selecting patients for referral for suspected cancer is a complex procedure which is influenced by 1) the general practitioner’s (GP’s) personal characteristics such as knowledge, skills, clinical judgement, risk-taking disposition and attitude; 2) organisational factors such as access to tests and investigations; and 3) patient-related factors such as expectations, symptom presentation and communication-style preferences. It is by no means a straightforward task to set the bar for referral [12]. Over-investigation has clinical and financial consequences, whereas under-investigation may delay diagnosis and have medico-legal implications.

The key role of general practice in earlier cancer diagnosis is indisputable; 85% of Danish cancer patients presented symptoms to general practitioners on their route to diagnosis [13]. Previous studies have found that cancer patients had an increased number of visits to general practitioners 6 months prior to diagnosis [14] and that 25% of all cancer patients had a primary care interval of more than 20 days until referral according to the GPs [15,16]. Thus, there seems to be a potential for earlier referral and possibly also for earlier diagnosis [17-21]. However, as the average Danish GP sees only eight to ten new cancer patients per year, cancer is a relatively rare disease in primary care. Half of the diagnosed cancer patients are known to present with what the GPs report as alarm symptoms, while the other half tend to present with non-specific or vague symptoms [13,15]. When patients present cancer-related symptoms to general practitioners, their probability of having cancer is low because even alarm symptoms of cancer have low positive predictive values (PPVs; below 5%) [22-30]. Yet, the proportion of cancer patients among patients referred to cancer fast-track pathways is high (PPVs of 10%–30%) [31,32], which implies that the GPs may somehow introduce a higher threshold for referral.

To improve cancer diagnostics in Denmark, the latest Danish Cancer Plan included continuing medical education (CME) targeting GPs as a key strategy to optimise cancer symptom recognition among GPs and to facilitate referral to fast-track pathways. The CME initiative provided an important opportunity to measure if and how targeted CME may influence the participating GPs’ knowledge, attitude and activity in the context of referral for cancer suspicion.

The primary aim of this article is to describe the conceptualisation, operationalisation and implementation of CME to enhance cancer diagnostics in primary care. A second aim is to describe the study design and outcomes chosen to evaluate the effects of the CME.

## Method

### The CME intervention

#### Setting

The study was performed in one of the five Danish Regions (Central Denmark Region) which has 1.27 million inhabitants and 417 GP clinics with 836 GPs. In Denmark, GPs own their private clinics and are on a contract with the Danish Regions on a central, negotiated scheme [33]. Part of this scheme includes remuneration for participation in CME. As a first line in the Danish healthcare system, the GPs provide free access to medical advice and act as gatekeepers to hospital and private specialists. Each citizen (99%) is listed with a specific general practice clinic.

The regional CME on early cancer diagnosis was initiated by a team comprising researchers, a regional academic coordinator, hospital-GP liaison advisors, a CME supervisor and three leading hospital consultants. A working group was established to operationalise the CME. This group counted four academic GPs of whom two were hospital-GP liaison advisors, one CME supervisor and one researcher (BST). A research group (the authors) identified the barriers as described below. The Danish government funded the CME.

#### Identification of barriers at GP level for early cancer diagnosis

**Figure 1:**
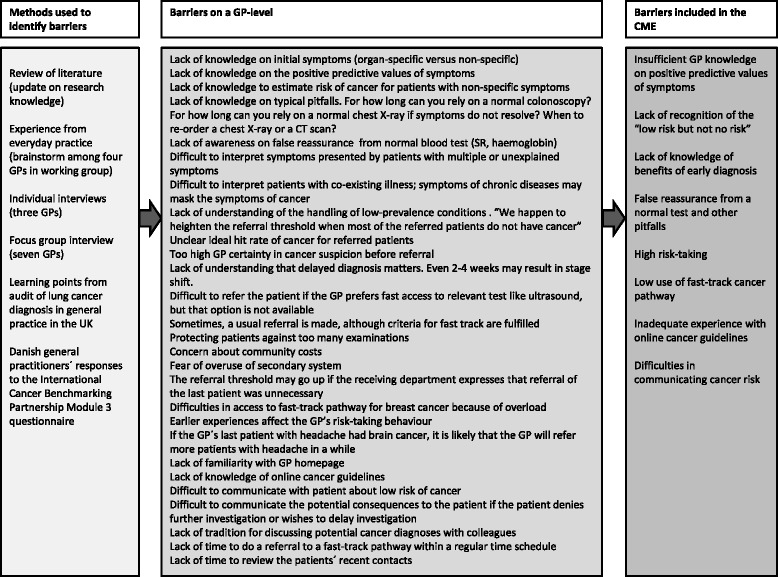
**The process of identifying the important barriers to be included in the CME program.**

The key aim of the CME was to optimise cancer-related referral from general practice clinic to hospital in an attempt to identify underlying cancers at an earlier and more amenable stage. The target for the intervention was the individual GP, and the CME was developed in a stepwise manner as described by Grol et al. [34]. Initially, the research group identified barriers at the GP level for early referral using a number of methods: input from research literature, brainstorm among the four GPs in the working group, individual GP interviews made by the first author (BST), a focus group interview with six GPs [35], experiences from audit of lung cancer diagnosis in general practice in the UK [36], and Danish GPs’ responses to the Module 3 International Cancer Benchmarking Partnership (ICBP) questionnaire [37]. The barriers identified through this process were shared with the members of the working group, and they were included in the CME if considered both important and possible to change (Figure 1).

One barrier was “Insufficient GP knowledge about the positive predictive values (PPV) of cancer symptoms”. This barrier signifies that GPs have not acquired full knowledge of the PPVs based on primary care populations [22-27,38]. It was hypothesised that this could prevent GPs from knowing what would be the PPV upon referral of patients to the fast-track cancer pathway (below 10%). Another barrier was “Lack of recognition of the ‘low-risk-but-not-no-risk’ symptoms”. This barrier reveals the absence of awareness of non-specific, vague symptoms [39,40]. “Lack of knowledge on benefits of early diagnosis” suggests an insufficient understanding of the fact that 2–4 weeks’ delay in diagnosis may cause stage progression for some cancers [41] and that waiting time in general can have an impact on prognosis [8,42]. Another important issue, “false reassurance from a normal test and other pitfalls”, covers the fact that GPs felt reassurance when an ordered test turned out normal or was used on mistaken indications. This reassurance could arise because of the false negative nature of the results (e.g. chest x-ray missing 15-20% of lung cancers [43]) or because a negative test result was used to exclude a cancer diagnosis (e.g. normal blood counts). Awareness of typical pitfalls [44] in early cancer diagnosis was thus considered important to the GP’s choice and interpretation of a test.

“High risk-taking” indicates the existence of a high GP referral threshold [45]. The threshold is considered to be too high since the observed proportion of cancer patients in Danish fast-track pathways (typically 10-30%) [31,32] is higher than the PPVs of alarm symptoms. “Low use of fast-track pathway referral for cancer suspicion” indicates that in some situations GPs refer patient to an ordinary hospital investigation rather than to the more optimal cancer fast-track pathway. This could be explained by an incomplete implementation or a concern for overuse of the fast-track referral option. “Inadequate experience with online cancer guidelines” indicates a need for more experience in the use of the regional website for referral. It was hypothesised that such a website would heighten the GPs’ knowledge about referral criteria and receiving departments and that the presence of this website would allow the GPs an opportunity to better inform the patient about the investigation and overall reduce time spent. “Difficulties in communicating cancer risk” concealed an uncertainty in how to communicate risk to a patient. This item was evaluated as important since, opposite ordinary referral, referral to a fast-track pathway required that the GP informed the patient about their suspicion of cancer. Thus, some GPs did not refer patients to cancer fast-track investigation as they did not want to worry the patient.

#### Addressing the identified barriers and developing the CME elements

According to the theoretical frame described by Michie et al. [46], any changes in the GP’s clinical behaviour (B) will be governed by their psychological and physical capability (C), social opportunity (O) and motivation (M) to change. Using this COM-B-model, the identified barriers were analysed, and the elements of the CME were subsequently developed (Table 1).

**Table 1:** **The operationalisation of the specific CME elements based on the identified barriers**

**Barriers**	**Capability Psychological/physical**	**Opportunity Social** **/physical**	**Motivation Automatic** **/reflective**
Insufficient GP knowledge of PPV of symptoms of cancer	Positive predictive values for cancer symptoms.	PPV cards followed by explanation were delivered as hand-outs to bring home to practice.	
Lack of recognition of the “low risk but not no risk” symptoms	Only 50% of cancer patients presented organ alarm symptoms.		Evoking emotions by watching the educational film. Case-based experience. Exchanging experience from daily practice.
Lack of knowledge of benefits of early diagnosis	Explaining the waiting paradox. Delay may influence prognosis.		Understanding the value of early diagnosis.
False reassurance from a normal test and other pitfalls	20% of lung chest X-rays are false negative. Only 33% of ovarian tumours can be found with a gynaecological examination. Blood test cannot exclude a risk of cancer.		
High risk-taking	Cancer is a low-prevalence condition in general practice. Even alarm symptoms have low PPVs for cancer. This explains the need for having a low PPV for cancer among referred patients.	Debate on pros and cons of early cancer diagnosis. Opinion leaders and hospital consultants backing up earlier referral.	Understanding the need for having a low PPV for cancer among referred patients. Debate on cost and overdiagnosis. Debate on the GP’s role
Low use of fast-track cancer pathway referral	Fast-track referrals reduce diagnostic intervals. If not, it is a challenge for the hospital to redirect patients referred via the usual route to a cancer fast-track pathway or the patient risks investigatory delay.	Fast-track pathways for cancer investigation already available. Increasing experience in using regional GP website raised awareness on referral guidelines.	Sharing experience. Accepting that 95% of referred patient will not have cancer.
Inadequate experience with online cancer guidelines	Reintroducing the regional GP homepage. Guiding in search strategy and contents, including referral guidelines.		Improving the GP’s self-confidence in decision-taking
Difficulties in communicating cancer risk	Most patients presenting alarm symptoms had been worried about cancer before consulting a general practitioner.	Patient leaflets were delivered to support communication about cancer risk.	Sharing experience on cancer risk communication.

The CME-elements were approached by capability, opportunity or motivation (COM).

The psychological capability is the GP’s ability to engage in the necessary thought processes related to comprehension and reasoning. The cognitive part of the CME would increase the GP’s knowledge about the PPVs of cancer symptoms and increase his or her awareness of the identified pitfalls in cancer diagnosis. This was believed to lead the GP to perform a more correct cancer risk assessment and thereby optimise referral procedures. The CME would point out that referral-delay could worsen the prognosis to dispel the myth that cancer develops over several years and that another month therefore would not change the prognosis [8,47].

The physical capability covers the skills necessary to perform concrete manoeuvres. The CME reintroduced how to access the regional website and shortly described the contents and search tool. Furthermore, the communication skills about cancer were addressed through case-based discussion on how and when to inform a patient about cancer risk.

The GPs’ physical opportunity to refer patients suspected of having cancer exists through the already established fast-track pathways and existing guidelines. The CME included no intervention in this area. The social opportunity was indirectly addressed by including a local opinion leader and a local hospital consultant in the team of lecturers to emphasise that a change of attitude towards lowering the referral threshold is widely recommended.

Motivation includes the reflective processes involving evaluation. Risk taking was addressed through case-based discussion and by raising questions about what it implies to be a competent GP. Motivation also comprised automatic processes involving emotions and impulses arising from associative learning. To address these processes, the CME included an educational film developed to illustrate a GP’s handling of a patient with a complex medical history. By identifying with the GP actor and by subsequently discussing his struggle and lack of readiness to do the definitive investigation, it was intended that the GPs would store this experience in their mind for later pattern recognition.

#### Form of CME

The CME took place as a 3-hour session. According to the available evidence, the teaching approach was multifaceted, partly didactic, but mostly interactive [48,49]. The key points were first explained and illustrated by an oral presentation, next they were discussed in a reflective debate based on two clinical patient scenarios and one educational film to create a scenario similar to that encountered in clinical practice and decision-making. Hands-on models of tumours were placed in the lecture room. PPV diagrams on lung, colorectal, ovarian and prostate cancer [22-27] were printed in hard copy as a clinical tool. Furthermore, leaflets for specific fast-track pathway to support communication about risk of cancer were offered in a plastic retainer, also to bring home to the clinic. The CME schedule is shown in Table 2.

**Table 2:** **The schedule for the 3-hour CME meeting provided in the eight clusters**

**Time (minutes)**	**Form**	**Lecturer**	**Barriers**	**Content**
10	Introduction	Regional academic coordinator		Presentation of schedule and lecturer
10	Plenary discussion	Hospital-GP liaison advisors, GP participants	Risk-taking, PPVs of symptoms	Questions regarding risk-taking and symptoms PPVs
10	Reflection, group discussion	GP participants	Risk-taking	
			PPVs of symptoms	
25	Power point presentation	Primary care researcher	Benefits of early diagnosis	GP role in cancer diagnosis, times to diagnosis matter
	Plenary discussion		Risk-taking	Early stage—vague symptoms
			Use of fast-track referral	Description of use of fast-track referral contra usual referral
10	Power point presentation	Hospital-GP liaison advisors	Use of fast-track referral	Audit on quality of referral letters and discharge letters in relation to cancer investigation
10	Power point presentation	Senior doctor from local hospital	Risk-taking	Cancer diagnosis at hospital level
	Plenary discussion		Use of fast-track referral	Cooperation with primary care
			Communicating risk	Always inform patients about cancer suspicion
10	Group discussion	GP participants		Debate: How are diagnostics done in your clinic?
10-min break (hands-on models were introduced)				
40	Educational film	Hospital-GP liaison advisors	Risk-taking	Educational film discussed in sequences
	Group discussion	GP participants	Use of fast-track referral	Awareness of barriers, pitfalls and difficulties in an everyday general practice setting.
	Plenary discussion		“Low risk but not no risk”	
			False reassurance/pitfalls	
			Communicating risk	
40-min break—dinner				
45	Power point presentation	Primary care researcher	PPVs of symptoms	Cancer patients’ symptoms in general practice
	Patient cases	Hospital-GP liaison advisors	“Low risk but not no risk”	Debate on patient cases
	PPV diagram		Risk-taking	Demonstration of use of PPV diagrams (handouts)
			False reassurance/pitfalls	
10	Power point presentation	Hospital-GP liaison advisors	Online cancer guidelines	Reintroduction of regional GP homepage.
			Communicating risk	Leaflets to support communicating cancer risk

#### Lecturers

The CME sessions were conducted by a team of six persons with different healthcare backgrounds. They consisted of an academic primary care researcher to increase the new evidence-based knowledge; two hospital-GP liaison advisors to reduce the gap between research knowledge and everyday life in practice; a local CME supervisor to establish a safe atmosphere and to facilitate acceptance of the knowledge by being considered a local opinion leader; a local hospital consultant to guarantee that the proposed changes could be anchored locally and to ensure a debate of any local issues that could act as barriers to the implementation. A regional academic coordinator introduced the program and ensured that time was kept.

#### Lecture room

The CME was intended to reach as many GPs as possible in the Central Denmark Region. Geographical accessibility was therefore important, and the CME sessions were accordingly arranged as eight local meetings. The meeting rooms had high-quality audio-visual equipment and were laid out with round tables for 6–8 people. Participants were offered catering during the meeting, and a friendly, relaxed atmosphere was established.

#### Piloting

A full-scale CME session was conducted with the Regional Cancer Steering group as audience. Feedback from the session was used to further adapt the CME.

### Study design

**Figure 2:**
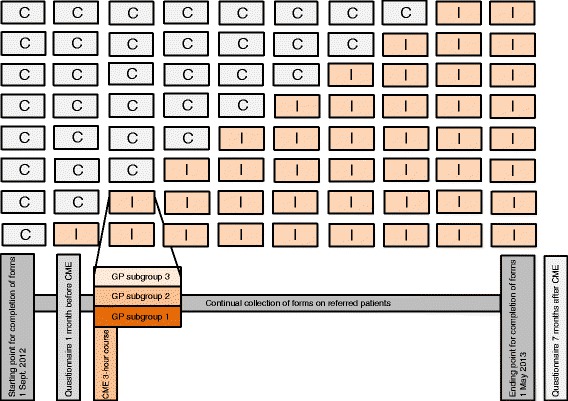
**The stepped wedge design to evaluate the effect of the CME.** The first time point to the left indicates the baseline measurement where all of the clusters were controls (C). At subsequent time points, clusters received the CME intervention and crossed over to interventions (I). All clusters eventually received the intervention. The bottom of the figure focuses on one cluster to illustrate the data collection and the three subgroups for stratification: 1) GPs who participated in the CME; 2) GPs who did not participate in the CME, but who had at least one colleague who participated; 3) GPs who did not participate in the CME and who had no colleagues who participated.

The effect of the CME intervention will be evaluated in a geographical cluster randomised stepped wedge design [50]. All GPs in the Central Denmark Region were allocated to one of eight clusters based on their clinics’ geographical location. CME was offered as a 3-hour meeting to each cluster with 3 weeks’ interval in between clusters. Clusters with delayed intervention will serve as controls for clusters that have already received interventions (Figure 2).

The Regional Cancer Care Unit generated the random allocation sequence which was based on the pragmatic principle that dates for CME should be applicable for that geographical area (taking into account other arrangements in the area, the availability of venue, etc.). The same independent administrative unit enrolled and assigned clusters to the CME. The researchers were not involved in the randomisation. The GPs were not aware of the interventional design. The main researcher was present at the meeting, but only to register participants and to observe that the CME was delivered as planned to evaluate uniformity. She could not interfere with the participants or the CME conduct.

#### Study population and recruitment of GPs to the CME

All GPs from the Central Denmark Region were invited to the CME. The CME was announced in each cluster by mail invitations 2 months before and again 1 month before the CME. Additionally, the description of the meeting was available on the regional GP website. Each GP willing to participate in the CME was registered at sign-up and again upon arrival at the CME.

#### Inclusion and exclusion criteria for patients

Included are all patients referred from general practice to the fast-track pathway for cancer except patients suspected for non-melanoma skin cancer. Excluded are patients already diagnosed with cancer within five years prior to the date of referral.

### Outcomes

#### The primary outcomes

1. Primary care interval is defined as the time from the first presentation of a cancer-relevant symptom in primary care until the date of referral to secondary care for investigation [16]. It was hypothesised that the CME would lower the primary care interval by increasing the GP’s readiness to refer.
2. GP referral rate is defined as the number of patients referred by a specific GP to a cancer fast-track pathway per 1000 patients listed with the GP. The CME was expected to increase the referral rate.

#### The secondary outcomes

**Figure 3:**
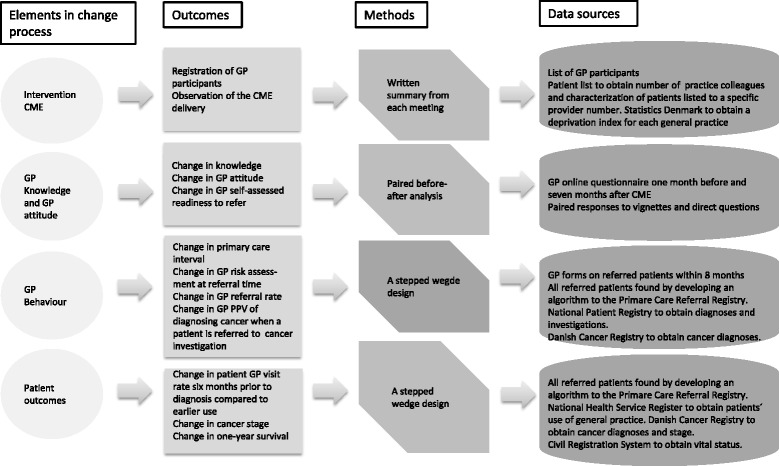
**The aspects of the interventions that were measured and the outcomes.** The first column depicts the elements in the change process; the second column classifies the corresponding outcomes; the third column describes the methods applied; and the fourth column depicts the data sources.

1. GP knowledge is defined as knowledge on *i)* Cancer as a condition with a low prevalence, *ii)* PPVs for cancer of selected symptoms, *iii)* Typical pitfalls and *iii)* Initial cancer presentation. This is measured by requesting the GP to indicate a probability percentage (0-100%) on questions such as; *what is the likelihood of a 50-year-old patient having cancer at the time you choose to refer to a cancer fast-track pathway? What is the likelihood of a patient, smoker, aged 40 and above, having lung cancer the second time the patient presents with haemoptysis in your practice? What is the risk for a lung cancer not being detected on a chest x-ray at the time of diagnosis?* W*hat is the proportion of patients with colorectal cancer who initial presented an alarm symptom*?
2. GP attitude towards early cancer diagnosis, assessed as a score on a 1–5 Likert scale. It is defined as attitude to *i)* Use of fast-track referral, i.e. whether the referral option is considered complicated and/or time-consuming. *ii)* Use of healthcare resources, i.e. whether risk taking is affected by fear of overuse or fear of delayed diagnosis. *iii)* Use of patient resources, i.e. whether risk taking is affected by fear for unnecessary distress or need for necessary investigation to find those with cancer in an early stage.
3. GP self-assessed readiness to refer is defined as the proportion of hypothetical patients referred at their first presentation based on GP responses to patient scenarios (vignettes) concerning cancer diagnosis. 4) GP-assessed risk of cancer for referred patients. 5) Referred patients’ GP visit rates 6 months prior to diagnosis compared with their earlier use of general practice. 6) The proportion of patients with cancer among referred patients for each GP (GP’s PPV for diagnosing cancer when a patient is referred to the cancer fast-track pathway). 7) Cancer patients’ clinical tumour stage at the time of initiating treatment. 8) Cancer patients’ one-year survival measured as the proportion of cancer patients per GP alive one year after the date of diagnosis (Figure 3).

### Data

Data are collected by GP-completed online questionnaires before and after the CME and by GP-completed one-page forms on each patient referred to cancer investigation within an 8-month period surrounding the CMEs. These data will be expanded with register data.

#### Online questionnaire: Development, pilot testing and data collection

The online questionnaire consisted of vignettes and direct questions. The vignettes were validated and tested in the ICBP module-3 studies [37]. They were developed to describe the likely GP actions which included GP readiness to refer when a patient presented with symptoms suggestive of lung cancer (two vignettes), colorectal cancer (two vignettes) or ovarian cancer (one vignette). GPs were asked to complete two randomly chosen vignettes. Each vignette had two or three phases: phase one represented the first patient presentation; phases two and three represented subsequent visits where the patient’s symptoms had developed; each phase had a defined PPV for cancer.

The direct questions were based on the elements of the CME developed as *ad hoc* questions to measure a possible CME effect on knowledge (6 items) and attitude (13 items). The first version of the questionnaire was developed by the authors and academic GPs in an iterative process that focused on the specific elements for CME intervention. The questionnaire was piloted for cognitive understanding among six doctors and, finally, among 12 GPs. Data quality, missing variables, and ceiling and floor effects were checked, and a revised version of the questionnaire was produced (Additional file 1).

The GPs were allocated a CME date. The invitation to the online questionnaire was sent by e-mail to the GPs 1 month before the CME. Non-responders received a reminder by e-mail after two weeks. A second questionnaire similar to the first one except for two new randomly selected vignettes was sent by e-mail 7 months after the CME to GPs who responded to the first questionnaire. Non-responders were reminded by e-mail after two weeks.

#### One-page forms on referred patients: Development, pilot testing, and data collection

This form was used to collect specific clinical information on patients referred from GPs to the fast-track pathway on cancer suspicion. The one-page form requested the patient’s personal identification number, the date of hospital referral, the date of first presentation of symptoms in general practice related to cancer suspicion, the symptoms presented at the time of referral, the specific fast-track pathway, and the GP’s assessment of the patient’s risk of cancer. GPs were asked to rate a specific risk for cancer by indicating a probability percentage (0–100) (Additional file 2).

All the items mentioned above have previously been validated in studies performed by the Research Unit [15,44]. The form was piloted in three steps. Seven GP trainees were observed while they filled in the form about the most recent patient they had referred on cancer suspicion. They were interviewed about any problems encountered and how they understood the items. Three GPs then completed forms in their daily practice over a 2-week period in order to check its feasibility. Finally, 21 GPs were asked to fill out and subsequently send the forms electronically to the Research Unit to verify the feasibility of the electronic transfer and the optical scanning of the forms [51].

In August 2012, all GPs in the Central Denmark Region received a pad with 25 forms. They were requested to complete a form each time they referred a patient to a cancer fast-track pathway within an 8-month period (September 2012-May 2013). Patients referred to fast track were also identified in a regional database (PAS) and in a radiological database (breast cancer). If the GP form was missing, the GP was sent a form for the particular patient and kindly requested to complete it. This form was extended with an item on the patient’s route into a fast-track pathway investigation to include only patients referred from GPs on cancer suspicion (Additional file 3).

The one-page form was designed and processed in the computer programme Teleform Enterprise Version 8.0 (Cardiff software Inc., San Marcos, CA, USA). Handwritten data including presenting symptoms were coded by the first author (BST) before scanning [51].

All data will be transferred to the statistical program Stata (Stata software, version 11.1, StataCorp, College Station, Tex, USA) and will be checked for errors. If errors are encountered, the original form of the completed questionnaire will be inspected, and the database entry will be corrected.

#### Incentives

Remuneration was 33 Euros for completing the first online questionnaire and 17 Euros for completing the second online questionnaire. The GPs received 100 Euros for completion of forms on referred patients during the 8-month period.

#### Register data

Each inhabitant with a permanent residence in Denmark has a 10-digit personal identification number, the CPR number. This number is registered in the Danish Civil Registration System and allows linkage between all national registries at the individual level. A cancer patient’s vital status will be obtained 12 months after the date of diagnosis.

The provider number registry contains the names and addresses of every Danish GP, the number of GPs in each general practice, and patient list size including the patients’ sex and age.

The Primary Care Referral Registry (an online database) stores copies of all electronic referral letters sent from general practice to hospital. We will develop an algorithm to identify patients referred on cancer suspicion in the 8-month study period to increase the completeness of the data on referred patients.

The Danish National Patient Registry [52] will be used to obtain information about comorbidity and cancer diagnoses.

The Danish National Health Service Registry [53] will be used to count the number of contacts of referred patients to general practice during the 6 months preceding the referral date. Each patients previous use of general practice patient will be counted and serve as a basis for comparison.

The Danish Cancer Registry [54] will be used to identify patients with cancer among all referred patients. From this registry, we will also obtain data on date of diagnosis, cancer type and tumour stage.

Statistics Denmark [55] holds data stored in the Integrated Database for Labour Market Research. These data will be used to calculate a deprivation score for each general practice (Table 3).

**Table 3:** **Detailed description of registers used for data collection to investigate the effect of the CME**

**Name of register**	**Description of registers**
The CPR number and the Danish Civil Registration System (CRS)	In Denmark (and other Nordic countries), researchers have exceptional opportunities to perform register-based research because every person with a permanent residence in Denmark has a unique personal identification number. At birth or immigration, all citizens in Denmark are allocated a personal ten-digit identification number, the CPR number. This number is registered in the Danish Civil Registration System (CRS) and allows linkage between all national registries at the individual level. The CRS contains information about vital status (dead or alive) and residence.
The Primary Care Referral Registry	An online shared database with all electronic referral letters sent from general practice clinics to hospitals. Contains information about sender, receiver and patient including anamnesis. We will develop an algorithm to identify patients referred for cancer investigation.
The Danish National Patient Registry(NPR) comprises The Patient Administrative System (PAS)	The NPR is a national database unifying information from the five regional Patient Administrative System (PAS). The NPR is run by the National Board of Health who carries out ongoing validation of the data from PAS. Both the NPR and PAS are continuously updated. They comprise variables like the patient CPR number, dates of admission and discharge, diagnoses classified according to the International Classification of Diseases (ICD-10) (Comorbidity), codes for undertaken procedures, the GP’s provider number and different additional codes. Of particular relevance for this study, we identified patients referred to fast-track cancer pathways by the additional code DZO31 in PAS every second week.
List of patients referred to fast-track pathway for breast cancer from four radiological departments	Since patients referred to the fast-track pathway for breast cancer are not registered with a DZO31 code, like other investigations in fast-track pathways, an additional data list was periodically received from the four radiological departments in Central Denmark Region.
The Danish Cancer Registry (DCR)	The DCR has been recording primary cases of cancer on a nationwide basis since 1943 and has been shown to be accurate and to have a nearly complete registration of cancer cases. The files of the DCR provide information on cancer type, site morphology and history of cancer. Tumours in the registry are coded according to the ICD-10 (seventh revision before 2004), which includes a four-digit code for tumour morphology. If a person develops more than one primary tumour, each tumour is generally registered and counted as an individual record. We used the DCR to find prior and incident cancer diagnoses among referred patients and to obtain information on date of diagnosis and tumour stage.
Danish National Health Service Registry (NHSR)	The NHSR for primary care is a national register of all health professionals contracted with the tax-funded health-care system, e.g. GPs. The register is run by the National Board of Health, and its data are based on the health professionals’ invoices to the regional health administrations. Among others, the NHSR holds information on name and addresses of every provider number. A provider number may refer to several providers if, for example, several GPs form a medical practice partnership.
Patient list	List of patients connected to each provider number. The list contains information on patients’ CPR number, including age and sex.
The provider number and the Provider Number Registry	Every health professional contracted with the tax-funded health-care system has a provider number. The provider number system is used to control the supply of GPs and, to a certain extent, to control expenditures. GPs are allowed to sell or share their provider number and office facilities. GPs can choose to work in solo practices or in group practices (in the latter case, the GPs can share a provider number or have one provider number per GP). Danish citizens are free to choose their own GP unless the GP list is closed (GPs are allowed to close their lists when the number of persons on the list reaches 1,600 persons). The list system enables the GP to develop a better knowledge of the individual patient which ensures continuity of care. The Provider Number Registry contains information on the name and addresses of every health professional with a provider number.
Statistics Denmark	As a central authority, Statistics Denmark is responsible for collecting, processing and publishing statistical information and for making statistical analyses and prognoses. Researchers can apply for data from Statistics Denmark for further analysis. We will use data from the Integrated Database for Labour Market Research which is owned by Statistics Denmark to calculate a deprivation score for each GP’s practice population. This Danish deprivation index (DADI) has eight variables that are scored individually and sum up to a score between 10 and 100; the higher the number, the greater the extent of deprivation in the practice population. The variables used are (i) proportion of adults aged 20–59 with no employment, (ii) proportion of adults aged 25–59 with no professional education, (iii) proportion of adults aged 25–59 with low income, (iv) proportion of adults aged 18–59 receiving public welfare payments (transfer income or social benefits), (v) proportion of children from parents with no education and no professional skills, (vi) proportion of immigrants, (vii) proportion of adults aged 30+ living alone and (viii) proportion of adults aged 70+ with low income (= the lowest national quartile).

### Statistical analyses

For the descriptive part, differences will be tested using appropriate parametric and nonparametric statistics. The effect of the intervention will be analysed in a generalised, linear, random-effects model with random effect of GPs. The data will be used to assess whether further modelling of inter-correlation within practice and within clusters is required, and whether the intervention effects are equal for all GPs or in random interaction with them. Analyses will be performed both in the full GP population “intention to intervene” (cluster level) and in the three GP subgroups: 1) GPs who participated in the CME, 2) GPs who did not participate in the CME, but had at least one colleague who participated, and 3) GPs who did not participate in the CME and who had no colleagues who participated. A colleague is defined as a GP with the same clinic address as the respondent. The intervention effect on GP knowledge and attitude will be measured in a before-after analysis.

#### Power calculation

Power calculation is based on the primary outcome: Primary care interval. The sample size is calculated to detect a reduction in the longest primary care intervals >20 days [56] from 25% to 20%. The expected effect is reduced according to an intervention to intervene analysis; we expect a GP participation rate at the CME-meetings of 40%. Uncorrected for clustering and repeated measures, a total sample size (N) of 5116 patients would be required (power of 99% and significance level of 0.05).

To accommodate the stepped wedge clustering effect N has to be multiplied by the design effect (DE) [57]: N_*sw*_ = DE*N, where

$$ DE=\frac{1+\rho \left(ktn+bn-1\right)}{1+\rho \left(\frac{1}{2}ktn+bn-1\right)}\cdotp \frac{3\left(1-\rho \right)}{2t\left(k-\frac{1}{k}\right)} $$

Here, *ρ* is the intra-cluster correlation, *k* is the number of steps, *n* is the average cluster size, *b* is the number of baseline measurements and *t* is the number of measurements after each step. In our study *k* = 8, *b* = *t* = 1. Furthermore, we assume that *ρ* = 0.05, as it is a typical value for intra-cluster correlation for clinical outcomes in primary care [58]. The cluster size can be expressed as *n* = *N*_sw_ / *c*, where *c* is the number of clusters. The number of clusters is known in advance (*c* = 8) and the question is how many subjects per cluster should be sampled to provide the power required. Using the equations described above, this cluster size is estimated as *n* = 200 through an iterative procedure. Therefore, the total sample required (*N*_sw_) is 1,653. In Central Denmark Region, 5,400 new cancer patients are expected in an 8-month period. We assumed that 40% [59] of those (2,200) will be referred from general practice to a cancer fast-track pathway. With an assumed PPV for diagnosing cancer in fast-track pathway on 20% and an assumed GP response rate on 70%, we expect to receive one-page forms with information on primary care intervals on 7,700 patients. Thus, it is considered that the study has ample strength—also in relation to three stratified subgroups of GPs and adjustment for multiple predictors.

### Ethical approval

The Danish Research Ethics Committee (j.no. 10/2014) concluded that no approval is necessary. The study was approved by the Danish Data Protection Agency (j.no. 2009-41-3471). The Danish National Board of Health gave legal permission to obtain information from the GPs’ medical records without permission from the patients (j.no. 3-3013-149/1/HKR). The regional Data Protection Agency gave permission to use the regional database (PAS) and the radiological database to obtain information on patients investigated in a cancer fast-track pathway (j.no. 1-16-02-262-12). The study is registered as NCT02069470 on ClinicalTrials.gov.

### Trial status

The study is ongoing. The CME meetings were conducted in the period September 2012 to May 2013. We have not yet conducted data cleaning or obtained data from registers; only the data from forms and questionnaires have been collected. Furthermore, we have not yet developed the algorithm to identify referred patients from the Primary Care Referral Registry.

## Discussion

We developed a multifaceted CME in early cancer diagnosis targeting the individual GPs using a step-wise approach for the development of complex interventions [34]. We identified key elements and analysed them by using the COM-B system described by Michie et al. [46]. The effect evaluation will be conducted in a geographical cluster randomised stepped wedge study. The effect of the CME will be measured at three levels: GP knowledge and attitude, GP activity and patient outcomes.

### Strengths and limitations of the study

With a detailed description of the CME elements, training process and context, we have tried to optimise the possibilities for an adaptation/replication in other settings.

The time frame of the present study did not afford us with the possibility to conduct an explorative study on the target group before the trial; instead, a full-scale CME was conducted in an external regional cancer steering group, and the findings were subsequently evaluated, discussed and approved.

Randomising individual GPs to the CME intervention was considered unfeasible as much of the effect would include GPs working together and discussing cancer diagnosis within practices and in the CME groups. Preventing GPs from participation was considered unethical since the CME was a part of the Danish Cancer Plan, and it was considered important that all patients could benefit from an improved clinical practice. A cluster randomised stepped wedge study was thus preferred. Using this design, we will be able to control for time trends by allowing contemporaneous comparisons across clusters at different time periods.

Designing a study in real life, however, raised some challenges with regard to controlling the intervention. A specific CME date was offered to all GPs, but they were free to decide whether they wanted to participate or not. The collected data will allow us to compare the three GP subgroups of each cluster at a baseline level to explore potential selection bias.

GP knowledge, attitude and self-assessed readiness to refer will be measured by online questionnaires before and after the CME to allow paired analyses to be performed. The GPs were informed in the invitation letter that the questionnaire attempted to measure activity related to cancer diagnosis in general practice. This could have influenced the responses.

By placing a pad with forms in each GP office and by sending regular reminders (every second week), we expect to collect prospective data, i.e. to obtain data before the GPs know whether the referred patient had cancer or not. In cases where the GP forgot to complete a form and had to be requested to do so, information bias can occur. Furthermore, lead time bias will be considered when reporting the 1-year survival. This outcome will be evaluated in close relation to potential change in cancer stage.

## Conclusions

We have developed a theory-based CME in early cancer diagnosis. We have described the CME to allow our intervention to be replicated. We have outlined the geographical cluster randomised stepped wedge design chosen for measuring the effect of the CME. If the CME has an impact on the chosen outcomes, the model used for its development may be used in similar primary health care setting.

## Additional files

Additional file 1:
**The** ***ad hoc*** **questions developed to measure a possible CME effect on GP knowledge and GP attitude.**
Additional file 2:
**One-page form on a referred patient.**
Additional file 3:
**Reminder: One-page form on a referred patient.**

## Abbreviations

GP: General Practitioner
CME: Continuing Medical Education
COM-B System: A behavioural system involving three essential conditions, capability, opportunity and motivation
PPV: Positive predictive value
ICBP: International Cancer Benchmarking Partnership
CRS: Danish Civil Registration System
NPR: The Danish National Patient Registry
HSR: The Danish National Health Service Registry
DCR: The Danish Cancer Registry
PAS: Patients administrative system

## References

[1] Coleman M, Forman D, Bryant H, Butler J, Rachet B, Maringe C, Nur U, Tracey E, Coory M, Hatcher J, McGahan CE, Turner D, Marrett L, Gjerstorff ML, Johannesen TB, Adolfsson J, Lambe M, Lawrence G, Meechan D, Morris EJ, Middleton R, Steward J, Richards MA, ICBP Module 1 Working Group (2011). Cancer survival in Australia, Canada, Denmark, Norway, Sweden, and the UK, 1995–2007 (the international cancer benchmarking partnership): An analysis of population-based cancer registry data. Lancet.

[2] Butler J, Foot C, Bomb M, Hiom S, Coleman M, Bryant H, Vedsted P, Hanson J, Richards M, ICBP Working Group (2013). The international cancer benchmarking partnership: An international collaboration to inform cancer policy in Australia, Canada, Denmark, Norway, Sweden and the United Kingdom. Health Policy.

[3] Ferlay J, Steliarova-Foucher E, Lortet-Tieulent J, Rosso S, Coebergh JW, Comber H, Forman D, Bray F (2013). Cancer incidence and mortality patterns in Europe: Estimates for 40 countries in 2012. Eur J Cancer.

[4] Walters S, Maringe C, Butler J, Rachet B, Barrett-Lee P, Bergh J, Boyages J, Christiansen P, Lee M, Wärnberg F, Allemani C, Engholm G, Fornander T, Gjerstorff ML, Johannesen TB, Lawrence G, McGahan CE, Middleton R, Steward J, Tracey E, Turner D, Richards MA, Coleman MP, ICBP Module 1 Working Group (2013). Breast cancer survival and stage at diagnosis in Australia, Canada, Denmark, Norway, Sweden and the UK, 2000–2007: A population-based study. Br J Cancer.

[5] Walters S, Maringe C, Coleman MP, Peake MD, Butler J, Young N, Bergström S, Hanna L, Jakobsen E, Kölbeck K, Sundstrøm S, Engholm G, Gavin A, Gjerstorff ML, Hatcher J, Johannesen TB, Linklater KM, McGahan CE, Steward J, Tracey E, Turner D, Richards MA, Rachet B, the ICBP Module 1 Working Group (2013). Lung cancer survival and stage at diagnosis in Australia, Canada, Denmark, Norway, Sweden and the UK: A population-based study, 2004–2007. Thorax.

[6] Maringe C, Walters S, Rachet B, Butler J, Fields T, Finan P, Maxwell R, Nedrebø B, Påhlman L, Sjövall A, Spigelman A, Engholm G, Gavin A, Gjerstorff ML, Hatcher J, Johannesen TB, Morris E, McGahan CE, Tracey E, Turner D, Richards MA, Coleman MP, ICBP Module 1 Working Group (2013). Stage at diagnosis and colorectal cancer survival in six high-income countries: A population-based study of patients diagnosed during 2000–2007. Acta Oncol.

[7] Maringe C, Walters S, Butler J, Coleman MP, Hacker N, Hanna L, Mosgaard BJ, Nordin A, Rosen B, Engholm G, Gjerstorff ML, Hatcher J, Johannesen TB, McGahan CE, Meechan D, Middleton R, Tracey E, Turner D, Richards MA, Rachet B, ICBP Module 1 Working Group (2012). Stage at diagnosis and ovarian cancer survival: Evidence from the international cancer benchmarking partnership. Gynecol Oncol.

[8] Torring ML, Frydenberg M, Hansen RP, Olesen F, Vedsted P (2013). Evidence of increasing mortality with longer diagnostic intervals for five common cancers: A cohort study in primary care. Eur J Cancer.

[9] Neal RD (2009). Do diagnostic delays in cancer matter?. Br J Cancer.

[10] Probst HB, Hussain ZB, Andersen O (2012). Cancer patient pathways in Denmark as a joint effort between bureaucrats, health professionals and politicians-A national Danish project. Health Policy.

[11] Dyrop HB, Safwat A, Vedsted P, Maretty-Nielsen K, Hansen BH, Jorgensen PH, Baad-Hansen T, Bünger C, Keller J (2013). Cancer patient pathways shortens waiting times and accelerates the diagnostic process of suspected sarcoma patients in denmark. Health Policy.

[12] O'Donnell CA (2000). Variation in GP referral rates: What can we learn from the literature?. Fam Pract.

[13] Hansen RP, Vedsted P, Sokolowski I, Sondergaard J, Olesen F (2011). General practitioner characteristics and delay in cancer diagnosis. A population-based cohort study. BMC Fam Pract.

[14] Christensen CG, Fenger-Grøn M, Flarup KR, Vedsted P (2012). Use of general practice, diagnostic investigations and hospital services before and after cancer diagnosis - a population-based nationwide registry study of 127,000 incident adult cancer patients. BMC Health Serv Res.

[15] Hansen RP, Vedsted P, Sokolowski I, Sondergaard J, Olesen F (2011). Time intervals from first symptom to treatment of cancer: A cohort study of 2,212 newly diagnosed cancer patients. BMC Health Serv Res.

[16] Weller D, Vedsted P, Rubin G, Walter FM, Emery J, Scott S, Campbell C, Andersen RS, Hamilton W, Olesen F, Rose P, Nafees S, van Rijswijk E, Hiom S, Muth C, Beyer M, Neal RD (2012). The Aarhus statement: Improving design and reporting of studies on early cancer diagnosis. Br J Cancer.

[17] Dommett RM, Redaniel MT, Stevens MC, Hamilton W, Martin RM (2012). Features of childhood cancer in primary care: A population-based nested case–control study. Br J Cancer.

[18] Emery JD, Shaw K, Williams B, Mazza D, Fallon-Ferguson J, Varlow M, Trevena LJ (2014). The role of primary care in early detection and follow-up of cancer. Nat Rev Clin Oncol.

[19] Hamilton W, Roobottom C (2011). Early diagnosis of cancer by imaging: The primary care perspective. Radiography.

[20] Hamilton W (2010). Cancer diagnosis in primary care. Br J Gen Pract.

[21] Hamilton W, Vedsted P (2011). Cancer and primary care: The clinical and research agenda. Br J Gen Pract.

[22] Hamilton W (2009). The CAPER studies: Five case–control studies aimed at identifying and quantifying the risk of cancer in symptomatic primary care patients. Br J Cancer.

[23] Hamilton W, Peters TJ, Round A, Sharp D (2005). What are the clinical features of lung cancer before the diagnosis is made? A population based case–control study. Thorax.

[24] Hamilton W, Round A, Sharp D, Peters TJ (2005). Clinical features of colorectal cancer before diagnosis: A population-based case–control study. Br J Cancer.

[25] Hamilton W, Sharp DJ, Peters TJ, Round AP (2006). Clinical features of prostate cancer before diagnosis: A population-based, case–control study. Br J Gen Pract.

[26] Hamilton W, Kernick D (2007). Clinical features of primary brain tumours: A case–control study using electronic primary care records. Br J Gen Pract.

[27] Hamilton W, Peters TJ, Bankhead C, Sharp D (2009). Risk of ovarian cancer in women with symptoms in primary care: Population based case–control study. BMJ.

[28] Hamilton W, Green T, Martins T, Elliott K, Rubin G, Macleod U (2013). Evaluation of risk assessment tools for suspected cancer in general practice: A cohort study. Br J Gen Pract.

[29] Hippisley-Cox J, Coupland C (2013). Symptoms and risk factors to identify men with suspected cancer in primary care: Derivation and validation of an algorithm. Br J Gen Pract.

[30] Hippisley-Cox J, Coupland C (2013). Symptoms and risk factors to identify women with suspected cancer in primary care: Derivation and validation of an algorithm. Br J Gen Pract.

[31] Guldbrandt LM, Rasmussen TR, Rasmussen F, Vedsted P: **Implementing direct access to chest computed tomography in general practice – method, adaption and outcome.** PlosOne, in press. 10.1371/journal.pone.0112162 PMC4226510 25383780

[32] Meechan D, Gildea C, Hollingworth L, Richards MA, Riley D, Rubin G (2012). Variation in use of the 2-week referral pathway for suspected cancer: A cross-sectional analysis. Br J Gen Pract.

[33] Pedersen KM, Andersen JS, Sondergaard J (2012). General practice and primary health care in Denmark. J Am Board Fam Med.

[34] Grol R, Wensing M, Bosch M, Hulscher M and Eccles M. **Theories on implementation of change in healthcare, in Improving Patient Care: The Implementation of Change in Health Care.** 2nd edition. Edited by Grol R, Wensing M, Eccles M, Davis D. Oxford: John Wiley & Sons; 2013

[35] Morgan DL, Bottorff JL (2010). Advancing our craft: Focus group methods and practice. Qual Health Res.

[36] Mitchell ED, Rubin G, Macleod U (2013). Understanding diagnosis of lung cancer in primary care: Qualitative synthesis of significant event audit reports. Br J Gen Pract.

[37] Rose PW, Hamilton W, Aldersey K, Barisic A, Dawes M, Foot C, Grunfeld E, Hart N, Neal RD, Pirotta M, Sisler J, Thulesius H, Vedsted P, Young J, Rubin G, The ICBP Module 3 Working Group (2014). Development of a survey instrument to investigate the primary care factors related to differences in cancer diagnosis between international jurisdictions. BMC Fam Pract.

[38] Jones R, Latinovic R, Charlton J, Gulliford MC (2007). Alarm symptoms in early diagnosis of cancer in primary care: Cohort study using general practice research database. BMJ.

[39] Nielsen TN, Hansen RP, Vedsted P (2010). Symptom presentation in cancer patients in general practice. Ugeskr Laeger.

[40] Hamilton W (2009). Five misconceptions in cancer diagnosis. Br J Gen Pract.

[41] Jensen AR, Nellemann HM, Overgaard J (2007). Tumor progression in waiting time for radiotherapy in head and neck cancer. Radiother Oncol.

[42] Richards MA, Westcombe AM, Love SB, Littlejohns P, Ramirez AJ (1999). Influence of delay on survival in patients with breast cancer: A systematic review. Lancet.

[43] Stapley S, Sharp D, Hamilton W (2006). Negative chest X-rays in primary care patients with lung cancer. Br J Gen Pract.

[44] Jensen H, Nissen A, Vedsted P (2014). Quality deviations in cancer diagnosis: Prevalence and time to diagnosis in general practice. Br J Gen Pract.

[45] Vedsted P, Olesen F (2011). Are the serious problems in cancer survival partly rooted in gatekeeper principles? An ecologic study. Br J Gen Pract.

[46] Michie S, van Stralen MM, West R (2011). The behaviour change wheel: A new method for characterising and designing behaviour change interventions. Implement Sci.

[47] Richards MA, Smith P, Ramirez AJ, Fentiman IS, Rubens RD (1999). The influence on survival of delay in the presentation and treatment of symptomatic breast cancer. Br J Cancer.

[48] Schichtel M, Rose PW, Sellers C (2013). Educational interventions for primary healthcare professionals to promote the early diagnosis of cancer: A systematic review. Educ Prim Care.

[49] Forsetlund L, Bjorndal A, Rashidian A, Jamtvedt G, O'Brien MA, Wolf F, Davis D, Odgaard-Jensen J, Oxman AD: **Continuing education meetings and workshops: Effects on professional practice and health care outcomes.** *Cochrane Database Syst Rev* 2009, Issue 2. Art. No.:CD003030 10.1002/14651858.CD003030.pub2 PMC7138253 19370580

[50] Hussey MA, Hughes JP (2007). Design and analysis of stepped wedge cluster randomized trials. Contemp Clin Trials.

[51] Jørgensen CK, Karlsmose B (1998). Validation of automated forms processing. A comparison of TeleformT with manual data entry. Comput Biol Med.

[52] Lidegaard O, Hammerum MS (2002). The National Patient Registry as a tool for continuous production and quality control. Ugeskr Laeger.

[53] Olivarius NF, Hollnagel H, Krasnik A, Pedersen PA, Thorsen H (1997). The Danish National Health Register. A tool for primary health care research. Dan Med Bull.

[54] Gjerstorff ML (2011). The Danish Cancer Registry. Scand J Public Health.

[55] **Statistics Denmark.** [www.dst.dk/en]

[56] Jensen H, Torring ML, Larsen MB, Vedsted P (2014). Existing data sources for clinical epidemiology: Danish cancer in primary care cohort. Clin Epidemiol.

[57] Woertman W, de Hoop E, Moerbeek M, Zuidema SU, Gerritsen DL, Teerenstra S (2013). Stepped wedge designs could reduce the required sample size in cluster randomized trials. J Clin Epidemiol.

[58] Machin D, Campbell MJ, Tan SB, Tan SH (2009). Sample Size Tables for Clinical Studies.

[59] Jensen H, Torring ML, Olesen F, Overgaard J, Vedsted P (2014). Cancer suspicion in general practice, urgent referral and time to diagnosis: A population-based GP survey and registry study. BMC Cancer.

