# Supplementary material for: A geographical cluster randomised stepped wedge study of continuing medical education and cancer diagnosis in general practice
Source: Implement Sci. 2014 Nov 7;9:159. doi: 10.1186/s13012-014-0159-z (PMC4229614; doi:10.1186/s13012-014-0159-z)
Supplement: Additional file 1: — The ad hoc questions developed to measure a possible CME effect on GP knowledge and GP attitude. [file 13012_2014_159_MOESM1_ESM.pdf]

Additional file 1: *Ad hoc* questions on items addressing attitude and knowledge.

1. **Your experience of referral to fast-track pathways**
   For each of the following statements, please select the option that best reflects your view.

	strongly disagree	disagree	neither agree nor disagree	agree	strongly agree
It is complicated to make a cancer fast-track referral					
The time it takes to make a cancer fast-track referral is appropriate					

	never	rarely	sometimes	often	always
A patient-doctor consultation involving a cancer fast-track referral can be carried out within 15 minutes					
I always provide the patient with written patient instructions when I make a cancer fast-track referral					
I refer patients in the usual way to the hospital instead of using a cancer fast-track referral, even if I suspect cancer					
I use information from the online cancer guideline for general practitioners when I make a cancer fast-track referral					

2. **Your attitude towards cancer diagnosis and your consideration**
   For each of the following statements, please select the option that best reflects your view.

	strongly disagree	disagree	neither agree nor disagree	agree	strongly agree
I consider it overuse of health care resources if I have referred a patient to a cancer fast-track pathway and the patient turns out not to have cancer					
I think that I subject the patients to unnecessary distress if I refer the patient to a cancer fast-track pathway and the patient turns out not to have cancer					
The more patients of those I refer to a cancer fast-track pathway are diagnosed with cancer, the better a doctor I am					
I am a good doctor when I can refer a patient to a cancer fast-track pathway based on a reasonable suspicion and thus am able to quickly clarify whether the patient has cancer or not					
I hesitate to order test when I risk to over-diagnose					

3. Your experience with communication about cancer suspicion

For each of the following statements, please select the option that best reflects your view.

	strongly disagree	disagree	neither agree nor disagree	agree	strongly agree
It´s difficult to mention risk of cancer to a patient with alarm symptoms.					
I feel well-equipped to communicate with the coordinators of the cancer fast-track pathway.					

4. The following questions are about probabilities

Enter for each question a percentage of your best estimate

	percentage
In your estimation, what is the likelihood that a 50-year-old patient having cancer at the time you chose to refer the patient to a cancer fast track pathway?<br>Enter a percentage (0-100 %):_______ %	
In your estimation, what is the likelihood of a patient, smoker, aged 40 and above having lung cancer the second time the patient presents hemoptysis in your practice?<br>Enter a percentage (0-100 %):_______ %	
In your estimation, what is the probability that a patient aged 40 and above having colorectal cancer the first time the patient presents constipation and weight loss in your practice?<br>Enter a percentage (0-100 %):_______ %	
In your estimation, what is the risk for a lung cancer not being detected on a chest x-ray at the time of diagnosis?<br>Enter a percentage (0-100 %):_______ %	
In your estimation, what is the proportion of patients with colorectal cancer who do present themselves with an alarm symptom?<br>Enter a percentage (0-100 %):_______ %	
In your estimation, what is the proportion of patients with ovarian cancer not being detected by doing a gynecological examination in general practice at the time of diagnosis?<br>Enter a percentage (0-100 %):_______ %	
