# Supplementary material for: A geographical cluster randomised stepped wedge study of continuing medical education and cancer diagnosis in general practice
Source: Implement Sci. 2014 Nov 7;9:159. doi: 10.1186/s13012-014-0159-z (PMC4229614; doi:10.1186/s13012-014-0159-z)
Supplement: Additional file 2: — One-page form on a referred patient. [file 13012_2014_159_MOESM2_ESM.pdf]

Additional file 2: One-page form. The GP was requested to complete a form each time the GP referred a patient to a cancer fast-track pathway

CaP Research Centre for Cancer Diagnosis in Primary Care

AARHUS UNIVERSITET

Please complete this form each time you refer a patient to a cancer fast-track pathway

Insert the date of referral of your patient to a cancer fast-track pathway:

day month year

☐☐☐☐☐☐

Enter your patient´s personal identification number:

day month year xxxx

☐☐☐☐☐☐ ☐☐☐☐

Enter the type of cancer fast-track pathway the patient is referred to:

____ ☐☐

Enter your degree of certainty in your choice of which cancer fast-track pathway you referred the patient to:

☐ Totally certain

☐ Fairly certain

☐ Little uncertain

☐ Totaly uncertain

Please elaborate____ ☐☐

On which symptoms did you refer the patients?

Symptom 1: ____ ☐☐☐

Symptom 2: ____ ☐☐☐

Symptom 3: ____ ☐☐☐

Insert the date of the day the patient first presented symptoms in general practice which according to your present knowledge raised your suspicion that the patient could have cancer:

day month year

☐☐☐☐☐☐

What is the risk for this patient having cancer?

(0 % = no risk, 100 % = absolutely certain)

☐☐☐ ☐ I don´t know
