# Supplementary material for: A geographical cluster randomised stepped wedge study of continuing medical education and cancer diagnosis in general practice
Source: Implement Sci. 2014 Nov 7;9:159. doi: 10.1186/s13012-014-0159-z (PMC4229614; doi:10.1186/s13012-014-0159-z)
Supplement: Additional file 3: — Reminder: One-page form on a referred patient. [file 13012_2014_159_MOESM3_ESM.pdf]

Additional file 3: One-page reminder form, used if we obtained information from register data about a patient investigated in a cancer fast-track pathway and we have not received a 1-page form from

CaP
Research Centre for
Cancer Diagnosis in Primary Care

AARHUS UNIVERSITET

Form on a patient investigated in a cancer fast-track pathway

From register data, we have been informed that a patient with the following personal identification number listed at your general practice has been investigated in a cancer fast-track pathway. We kindly request the referring general practitioner to complete this form.

1	1	1	1	1	1	1	1	1	1

Enter your full name or your project ID-number (this number appears on your pad):

____ ☐☐☐☐☐

Referral options:

☐ I have referred the patient to a cancer fast-track pathway

☐ I have referred the patient in the usual way to a hospital on cancer suspicion, but not directly to a cancer fast-track pathway

☐ I have referred the patient in the usual way to a hospital and did not suspect cancer

☐ The patient was admitted to hospital by emergency

☐ Suspicion of cancer was based on screening

☐ I have not been involved

Other ____ ☐☐

Enter the degree of certainty in your choice of cancer fast-track pathway:

☐ Totally certain

☐ Fairly certain

☐ Little uncertain

☐ Totaly uncertain

Please elaborate____ ☐☐

Insert the date of the day the patient first presented symptoms in general practice which according to your present knowledge raised your suspicion that the patient could have cancer:

day month year

What is the risk that this patient has cancer?

(0 % = no risk, 100 % = absolutely high risk)

			☐ I don´t know

Insert the date of referral of your patient to a cancer fast-track pathway:

day month year

On which symptoms did you refer the patient?

Symptom 1: ____ ☐☐☐

Symptom 2: ____ ☐☐☐

Symptom 3: ____ ☐☐☐
